# Elevation as a barrier: genetic structure for an Atlantic rain forest tree (*Bathysa australis*) in the Serra do Mar mountain range, SE Brazil

**DOI:** 10.1002/ece3.1501

**Published:** 2015-04-14

**Authors:** Talita Soares Reis, Maísa Ciampi-Guillardi, Miklos Maximiliano Bajay, Anete Pereira de Souza, Flavio Antonio Maës dos Santos

**Affiliations:** 1 Departamento de Biologia Vegetal, Instituto de Biologia, Universidade Estadual de Campinas – UNICAMPCP 6109, 13083-970, Campinas, SP, Brazil; 2 Centro de Biologia Molecular e Engenharia Genética, Universidade Estadual de Campinas - UNICAMPCP 6010, 13083-970, Campinas, SP, Brazil; 3 Departamento de Genética, ESALQ/Universidade de São Paulo - USP12418-900, Piracicaba, SP, Brazil

**Keywords:** Altitude, gene flow, genetic diversity, microsatellites, Rubiaceae

## Abstract

Distance and discrete geographic barriers play a role in isolating populations, as seed and pollen dispersal become limited. Nearby populations without any geographic barrier between them may also suffer from ecological isolation driven by habitat heterogeneity, which may promote divergence by local adaptation and drift. Likewise, elevation gradients may influence the genetic structure and diversity of populations, particularly those marginally distributed. *Bathysa australis* (Rubiaceae) is a widespread tree along the elevation gradient of the Serra do Mar, SE Brazil. This self-compatible species is pollinated by bees and wasps and has autochoric seeds, suggesting restricted gene dispersal. We investigated the distribution of genetic diversity in six *B. australis* populations at two extreme sites along an elevation gradient: a lowland site (80–216 m) and an upland site (1010–1100 m.a.s.l.). Nine microsatellite loci were used to test for genetic structure and to verify differences in genetic diversity between sites. We found a marked genetic structure on a scale as small as 6 km (*F*_ST_ = 0.21), and two distinct clusters were identified, each corresponding to a site. Although *B. australis* is continuously distributed along the elevation gradient, we have not observed a gene flow between the extreme populations. This might be related to *B. australis* biological features and creates a potential scenario for adaptation to the different conditions imposed by the elevation gradient. We failed to find an isolation-by-distance pattern; although on the fine scale, all populations showed spatial autocorrelation until ∼10-20 m. Elevation difference was a relevant factor though, but we need further sampling effort to check its correlation with genetic distance. The lowland populations had a higher allelic richness and showed higher rare allele counts than the upland ones. The upland site may be more selective, eliminating rare alleles, as we did not find any evidence for bottleneck.

## Introduction

The problem of speciation in tropical rain forests has long intrigued ecology researchers (Federov 1966). Tropical forests brought attention to the fact that closely related species occur side by side, challenging the ideas of speciation by geographic isolation (Federov 1966). Given a geographic barrier, gene flow interruption was recognized as the primary step toward reproductive isolation (Mayr 1963), and the evolutionary mechanisms behind it could be drift, natural selection, or both. In the absence of a geographic barrier, we would expect unrestricted gene flow and a homogeneous distribution of genotypes in a population. However, even without any discrete barrier, geographic distance may play a role in isolating populations, hampering the movement of alleles among them (Wright 1943; Hardy and Vekemans 1999). Thus, more distant populations are expected to have less genetic exchange than nearer populations, as a function of limited pollen and seed transport across space. This generates a spatial genetic structure resulting from local genetic drift. Therefore, the set of pollinators and seed dispersal vectors influence the degree of genetic isolation (Loveless and Hamrick 1984).

However, nearby populations with no discrete geographic barrier between them may also suffer from ecological isolation driven by habitat heterogeneity, which may promote population divergence by local adaptation and drift as well (Linhart and Grant 1996; e.g., Antonovics 1971; Misiewicz and Fine 2014). Misiewicz and Fine (2014) found evidence for ecological divergence in an Amazonian tropical tree, *Protium subserratum*, across a mosaic of soil types. They pointed out higher levels of genetic differentiation between adjacent populations in different soil types than between geographically distant populations in the same soil type.

In addition to soil type, other ecological features can interfere with the drift-gene flow balance across the landscape and produce a genetic structure within a species. In this context, elevation gradients, typical of mountain ranges, are special cases of landscape variation that influence both the genetic structure and the diversity of populations, particularly those located marginally at the upper and lower distributional limits (Herrera and Bazaga 2008; e.g., Byars et al. 2009; Shi et al. 2011). In this type of gradient, we can find a wide variation in environmental conditions over short distances, and the populations experiencing it could be subject to local adaptation (Byars et al. 2007; Shi et al. 2011). Factors such as temperature, precipitation, soil characteristics, and community composition vary sharply and probably affect habitat suitability for a species (Grubb and Whitmore 1966; Grubb 1977; Gentry 1988; Lieberman et al. 1996). Besides, elevation gradients might represent significant barriers to gene flow, hindering the movement of pollinators and seed dispersers (Schuster et al. 1989). Byars et al. (2009) attributed the low gene flow between high and low altitudes they observed for the Alpine grass *Poa hiemata* to the phenological separation along the altitudinal gradient, which led to reduced opportunities for insect pollinators. These authors found a stronger genetic structuring between altitudes within transects than between transects, even though the distances between transects were larger.

Thus, elevation gradients display a remarkable variation in the distribution patterns of genetic diversity within and between populations along them (Ohsawa and Ide 2008). Concerning within-population genetic diversity, while some studies have demonstrated diversity peaks on higher slopes (e.g., Gämperle and Schneller 2002), others have found greater diversity at lower (e.g., Quiroga and Premoli 2007) or intermediate elevations (e.g., Oyama et al. 1993). In addition to these three patterns, there are also reports showing constant values of genetic variability all over the gradient (e.g., Truong et al. 2007), suggesting a free gene flow among populations from different altitudes. The process behind all these patterns can be variable, but it seems that the optimal environmental conditions for a species play an important role in concentrating its major genetic diversity (Ohsawa et al. 2007; Ohsawa and Ide 2008). Plant populations in tropical forests are subject to varying levels of environmental heterogeneity, which ultimately influence their survival and reproduction, so the spatial distribution of individuals is mostly related to the spatial distribution of resources and conditions (Harper 1977). Thereby, given a geographical gradient of environmental conditions, a species local abundance might become higher when near its optimal environmental conditions, decreasing gradually as it departs from it (Brown 1984; Eckert et al. 2008).

*Bathysa australis* (A. St.-Hil.) Hook. f. ex K. Schum. (Rubiaceae) is a tropical tree endemic to the Brazilian Atlantic forest, widely distributed along the altitudinal gradient of the Serra do Mar mountain range, Southeast Brazil. For this reason, together with *B. australis* biological features, we believe this species may serve as a model organism to study the effect of altitude on genetic structure, and diversity. *B. australis* displays great phenotypic variability in leaf attributes between lowland and upland populations at this gradient (*personal observation*) and is mainly pollinated by bees and wasps (Freitas and Andrich 2013) with an autochoric seed dispersal mode, that is, no known vector disperses their seeds. Such characteristics may indicate a restricted gene flow between these extreme populations and the possibility of an ongoing local adaptation to contrasting environmental conditions.

The flowers of *B. australis* are protogynous, that is, their stigmas become receptive before the opening of anthers (Freitas and Andrich 2013). This lag between stigma reception and anther opening, associated with the earlier flowering of lower populations in the Serra do Mar mountain range (*personal observation*), could generate an upward pollen flow. Because the stigmas from a tree at a given altitude will be receptive earlier than pollen donors are available at the same altitude, the outcrossed pollen pool of this tree might be partly composed of migrant pollen from lower altitudes (Gauzere et al. 2013). This increased contribution from outside pollen to upper populations may enhance their genetic diversity and reduce selfing rates.

*Bathysa* species are usually distributed along forest slopes between 600 and 1100 m.a.s.l. (Germano-Filho 1999), where we can find typical Montane tropical forests (Veloso et al. 1991). Even though *B. australis* can be found both on higher and lower slopes (Oliveira-Filho and Fontes 2000), we observed that the highest estimated abundances (>10 indiv.ha^−1^) are on the upper slopes (e.g., Arzolla 2002; Leite and Rodrigues 2008; Padgurschi et al. 2011; Pereira 2011; Sanchez et al. 2013), while the lowest densities are recorded on the lower ones (*e.g*., Moreno et al. 2003; Assis et al. 2011; Campos et al. 2011; Gomes et al. 2011; Prata et al. 2011; Sanchez et al. 2013), with rare exceptions (e.g., Gomes et al. 2011; Ramos et al. 2011). For this reason, we believe that the conditions found on the higher slopes are more favorable to the spreading of *B. australis* populations than the conditions found on the lower slopes, which would affect the species’ genetic diversity.

In this study, we aimed to investigate within- and between-population genetic structure and diversity for *B. australis* at two extreme sites in the altitudinal gradient of the Serra do Mar mountain range, southeast Brazil. We addressed the following three questions: (1) Is there a fine-scale spatial genetic structure within *B. australis* populations, as would be expected, considering *B. australis* dispersal mode?; (2) On a larger scale, Is there a genetic structure between the upper and lower *B. australis* populations of the Serra do Mar mountain range?; (3) Are the upper mountain populations more genetically diverse than the lower ones?

## Materials and Methods

### Study species

*Bathysa australis* is a tree species of the Rubiaceae family that belongs to the lower strata of the forest, reaching up to 15 m in height. It is an endemic species of the threatened Atlantic rain forest, distributed predominantly in south and southeast Brazil (Germano-Filho 1999). Its flowering period is usually from December to April, and fructification occurs from February to June (Freitas and Andrich 2013). *Bathysa* displays terminal thyrsus inflorescences and its flowers are hermaphrodite, homostylous, and self-compatible (Freitas and Andrich 2013). In spite of its compatibility system, *Bathysa* flowers are dichogamous, a temporal separation of the male and female functions that may promote outcrossing in hermaphrodite flowers (Freitas and Andrich 2013). The main reward provided by their flowers is nectar, and the key pollinators are social bees and wasps (Freitas and Andrich 2013). The fruit is a capsule of 4–6 mm in length, and seed dispersal is autochoric (Zipparro et al. 2005; Colonetti et al. 2009).

### Study site and sampling design

**Figure 1:**
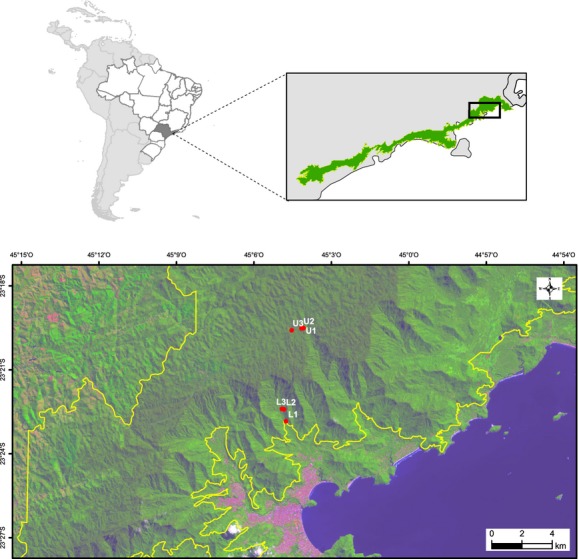
Map of the *Bathysa australis* populations sampled in the Serra do Mar mountain range. Location of L1, L2, and L3 lowland plots (Ubatuba, SP, Brazil), and U1, U2, and U3 upland plots (São Luis do Paraitinga, SP, Brazil), all within the limits of Serra do Mar State Park (yellow line). Image by Landsat 8 (654 RGB composition) taken in April 2014. Datum: WGS84 and 30 meters resolution.

This study was conducted at two sites along the elevation gradient (80–1100 m.a.s.l.) of the Atlantic forest of the Serra do Mar mountain range in São Paulo State, SE Brazil (Fig.1). Upland (1010-1100 m) and lowland (80–216 m) sites are within Serra do Mar State Park in the municipalities of São Luís do Paraitinga and Ubatuba, respectively. At both sites, *B. australis* populations were sampled in 1-ha plots, already established for the project “Biota Gradiente Funcional” (see Joly et al. 2012). In total, six populations were sampled, corresponding to six 1-ha plots: three at the lowland site (L1, L2, and L3) and three at the upland site (U1, U2, and U3).

The two sampled sites differ in climate and precipitation. While the lowland regional climate is tropical humid, with no dry season, the upland climate is subtropical humid (Setzer 1966). The lowland mean annual temperature is 22°C, and the average annual rainfall exceeds 2500 mm. Even in the driest months, from June to August, the average monthly precipitation is above 60 mm. The upland average annual temperature is 20°C and the average annual rainfall exceeds 1100 mm. In the driest months, from April to September, the average monthly precipitation is above 30 mm (EMBRAPA 2003).

According to the Brazilian National Classification System for Vegetation (IBGE System; Veloso et al. 1991), the upland sites are covered by montane dense ombrophilous forest and the lowland sites are covered by submontane dense ombrophilous forest. Indeed, these two sites differ markedly in vegetation structure and composition (Alves et al. 2010; Joly et al. 2012), but not in soil characteristics (Martins 2010).

A total of 1751 individuals (1044 at the lowland site and 707 at the upland site) were found in the six 1-ha plots, accounting for the whole population, that is, seedlings, juveniles, and adults (Table1). However, in each population, we collected leaf tissue only from *B. australis* individuals bearing reproductive structures that could reliably signal their reproductive status. Then, only 269 individuals were sampled in February and March 2012, 140 individuals at the lowland site and 129 at the upland site. The different sample efforts in each population reflect the abundance of reproductive individuals in each 1-ha plot at the time. These samples were placed in a thermos with ice and immediately frozen at −20°C for DNA conservation. In the laboratory, the samples were kept in a biofreezer at −80°C until DNA extraction.

**Table 1:** Genetic diversity for *Bathysa australis* populations using nine SSR polymorphic loci

Site	Population	Coordinates	Elevation range (m)	N sampled	N total	*A*	*A*_*e*_	*A*_*R*_	*H*_*o*_	*H*_*e*_	*F*
Lowland	L1	23°22′51.23″S	80–120	74	827	10.44 (1.42)	4.59 (0.65)	7.60 (2.4)	0.56 (0.08)	0.73 (0.05)	0.221 (0.10)
		45°4′45.23″W									
	L2	23°22′25.81″S	176–198	28	107	8.11 (0.81)	4.03 (0.47)	7.02 (1.9)	0.55 (0.09)	0.72 (0.04)	0.225 (0.12)
		45°4′50.56″W									
	L3	23°22′24.70″S	200–216	38	110	8.78 (1.36)	4.51 (0.73)	6.89 (2.7)	0.60 (0.08)	0.72 (0.04)	0.176 (0.10)
		45°4′55.03″W									
Upland	U1	23°19′31.83″S	1050–1100	29	197	7.44 (1.02)	3.20 (0.42)	6.45 (2.4)	0.53 (0.08)	0.64 (0.05)	0.198 (0.10)
		45°4′4.64″W									
	U2	23°19′31.59″S	1010–1040	73	366	9.22 (1.12)	4.22 (0.49)	6.87 (2.1)	0.60 (0.06)	0.72 (0.05)	0.186 (0.04)
		45°4′9.89″W									
	U3	23°19′36.02″S	1010–1040	27	144	7.11 (0.82)	4.12 (0.70)	6.45 (2.2)	0.62 (0.06)	0.70 (0.05)	0.103 (0.07)
		45°4′32.25″W									
Lowland								7.169	0.573	0.738	0.224
Upland								6.591	0.588	0.707	0.169

*N* sampled = number of individuals sampled; *N* total = total number of individuals in the 1-ha population plot; *A *= mean number of alleles (standard error); *A*_*e*_ = effective mean number of alleles (standard error); *A*_*R*_ = mean allelic richness (standard deviation); *H*_*o*_ = mean observed heterozygosity; *H*_*e*_ = mean expected heterozygosity; *F *= mean fixation index (standard error). Mean allelic richness across lowland populations was higher than mean allelic richness across upland populations (10,000 permutations; *P *=* *0.027).

### Laboratory analysis

We extracted genomic DNA from leaf tissue samples using the DNeasy Plant Mini Kit (QIAGEN, Valencia, CA). The genetic variation of *B. australis* populations was examined using nine polymorphic SSR loci. All the loci were developed specifically for *B. australis*, and primer-pair sequences and detailed procedures can be seen in Reis et al. (2013). PCR amplifications were performed in a 15-*μ*L volume containing 15 ng DNA, 1 × PCR buffer, 0.15 mmol/L each dNTP, 0.8 mmol/L each primer, 0.04% bovine serum albumin (BSA), 1.5 mmol/L MgCl2, and 1 U Taq DNA polymerase. A PTC-100 thermal cycler (MJ Research) was used with the following program: 96°C for 1 min, followed by 30 cycles of denaturation at 94°C for 1 min, 1 min at a specific annealing temperature, and a final extension of 72°C for 5 min. Amplified products were checked by electrophoresis on 3% agarose gels containing 0.1 mg ethidium bromide per milliliter in 1 × TBE buffer. The amplicons were electrophoretically separated using an ABI 3500 automated sequencer (Applied Biosystems, Foster City, CA, USA) with GeneScan 600 LIZ marker as the size standard (Applied Biosystems). Fragment size and allele identification were determined using the software GeneMarker version 2.2 (SoftGenetics, State College, PA).

### Linkage disequilibrium and null alleles

We tested for linkage disequilibrium (LD) using FSTAT with a Bonferroni correction for multiple comparisons. The presence of null alleles was checked using the software FreeNA (Chapuis and Estoup 2007).

### Descriptive statistics

Descriptive statistics were performed using GenAlEx version 6.5 (Peakall and Smouse 2006), with the following diversity parameters: number of alleles (*A*), effective number of alleles (*A*_*e*_), observed heterozygosity (*H*_*o*_), expected heterozygosity (*H*_*e*_), and fixation index (*F*). We also calculated allelic richness using the program FSTAT (Goudet 1995), because this parameter is more appropriate for comparisons among samples of different sizes (Leberg 2002). Hardy–Weinberg equilibrium (HWE) tests were conducted using GENEPOP version 4.2 (Raymond and Rousset 1995).

### Fine-scale spatial genetic structure

The fine-scale genetic structure was investigated in each population at the plot scale using the software SPAGeDi (Hardy and Vekemans 2002). We used the x,y coordinates of individuals in each 1-ha plot to generate a geographic distance matrix between pairs of individuals. The upper limits for our set of six distance classes were 10, 30, 50, 70, and 100 m. The pairwise estimated genetic distance was the kinship coefficient (*Fij*) by Loiselle et al. (1995), which does not assume HWE.

### Between-population genetic structure

The population structure inference as well as the number of existing genetic clusters and individuals assigned to each cluster was performed using the software STRUCTURE v.2.3 (Pritchard et al. 2000), which uses a Bayesian approach, the Markov Chain Monte Carlo (MCMC). STRUCTURE was run with different values for the number of clusters (*K*), varying from 1 to 7 under the admixture model, with no prior population information. To verify the robustness of our results, we performed 20 independent runs per *K* value with 200,000 burn-in periods and 500,000 Markov Chain Monte Carlo iterations. The statistics described by Evanno et al. (2005) was used to detect the most likely number of groups (*K*). The *K* value that best represents the structuring of populations can be identified by the peak value of Δ*K*.

We used a molecular variance analysis (AMOVA, Excoffier et al. 1992) to partition genetic variability between sites (elevational bands), between populations, and within populations. These estimates were made using the software GenAlEx 6.5 (Peakall and Smouse 2006). The combined effects of distance and altitude were checked by correlations of genetic differentiation with geographical distance and with altitudinal difference using Mantel tests and partial Mantel tests (Legendre and Legendre 1998). For Mantel tests, the significance of the correlation between two matrices was assessed by the permutation of rows and columns in the second matrix. The significance of the correlations between genetic distance, expressed as *F*_ST_, and geographic distance was estimated with 10,000 permutations. Mantel tests within altitudinal groupings were also carried out, but as an individual-based analysis, because of the low number of populations. We used the software PAST (Hammer et al. 2001) to calculate the Euclidean distances between individual pairs, and the software R (R Development Core Team 2008), package “vegan” (Oksanen et al. 2013), to perform Mantel and partial Mantel tests.

### Between-altitude genetic diversity

Diversity estimates for the upland and lowland sites were compared (by grouping samples from different sampling sites, either upland or lowland) after 10,000 permutations in FSTAT. Furthermore, we counted exclusive and rare alleles (frequency ≤ 0.05) in each locus to complement diversity comparisons between the upland and lowland sites. These comparisons were made with a Wilcoxon paired test in R (R Development Core Team 2008), as sample sizes among sites were very similar.

### Bottleneck

The software BOTTLENECK version 1.2.02 (Cornuet and Luikart 1996) was used to evaluate the hypothesis of historical reduction in effective population size, which would decrease genetic diversity (bottleneck). BOTTLENECK is based on the evidence that recently bottlenecked populations exhibit an excess of gene diversity (heterozygosity) among polymorphic loci. The expected heterozygosity is compared with the expected heterozygosity at mutation–drift equilibrium, given the allele number and the population sample size. If a population has undergone a bottleneck, gene diversity is higher than that expected at mutation–drift equilibrium, because the latter is calculated from the allele number, which drops faster than heterozygosity (Piry et al. 1999). Gene diversity was estimated under the two-phase model (TPM), setting 95% of the single-step stepwise mutation model (SMM) and 5% of the infinite allele model (IAM), with a variance of 12 among multiple steps, as recommended for microsatellite loci (Piry et al. 1999). Based on 1,000 replications, one-sided Wilcoxon signed rank tests (Luikart et al. 1998) were performed to evaluate whether the allele frequency distribution deviated significantly from the expected distribution under mutation-drift equilibrium.

## Results

### Linkage disequilibrium and null alleles

As we have found only one pair of loci showing LD in population L1 and another one in population U2, and the exclusion of these loci produced the same results, we kept them for our analyses. For each locus, the presence of null alleles was confirmed only in the populations of one of the altitudes. Considering that, we did not take any action, assuming that it could reflect a possible genetic structure between altitudes.

### Descriptive statistics

In the six populations sampled, the number of alleles per locus ranged from 2.0 to 16.0 (mean 8.5 ± 0.5). Allelic richness ranged from 2.0 to 11.1 (mean 6.9 ± 2.2). The observed heterozygosity ranged from 0.0 to 0.926 (mean 0.578 ± 0.03), and the expected heterozygosity in HWE ranged from 0.381 to 0.888 (mean 0.706 ± 0.02). The fixation index (*F*) fluctuated from −0.208 to 1.0, with a mean of 0.186 ± 0.04 and 72% of positive values. All populations had at least two loci with a significantly positive *F* value in the Hardy–Weinberg equilibrium test, indicating an excess of homozygotes. The locus BA30 from the L2 population, for example, did not show any heterozygous individual. All fixation index values were significantly positive using a 95% confidence level (*P* < 0.05), indicating deviations from HWE in all six populations (Table1).

### Fine-scale spatial genetic structure

**Figure 2:**
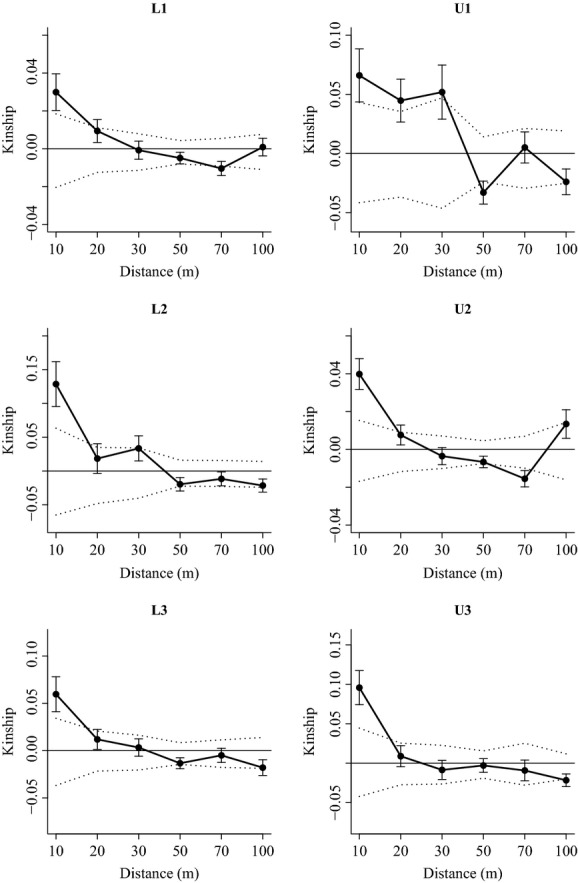
Fine-scale genetic structure for the six *Bathysa australis* populations. Average kinship coefficient as a function of geographical distance for each population analyzed. Bars indicate the standard deviation and dashed lines indicate the 95% confidence interval for the null hypothesis of no spatial genetic structure. Both lowland and upland populations exhibited autocorrelation in the first distance class.

On the local plot scale, all six populations have shown some degree of spatial genetic structure (Fig.2). Both lowland and upland populations exhibited autocorrelation in the first distance class, indicating that up to the range of ∼10–20 m nearby individuals are more genetically related than would be expected by chance.

### Between-population genetic structure

**Figure 3:**
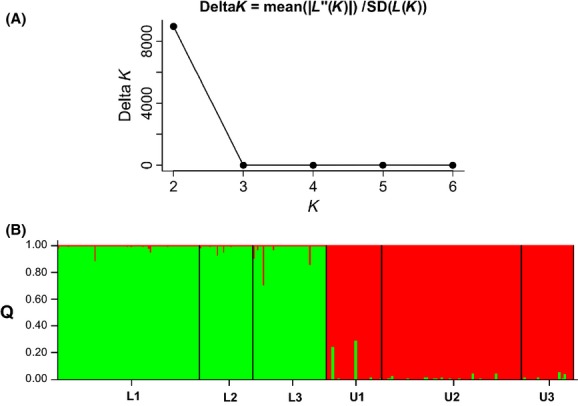
Genetic structure of *Bathysa australis* populations in the Serra do Mar mountain range. (A) Graphical plot based on delta-*K* calculated according to Evanno et al. (2005) to estimate the actual number of clusters for the 271 *Bathysa australis* individuals used in this study. (B) Assignment of 271 *Bathysa australis* individuals from six populations into two (*K *=* *2) clusters using a Bayesian-based population genetic structure analysis carried out with the software STRUCTURE (Pritchard et al. 2000). Each solid bar represents a single individual, while colored areas correspond to distinct genetic clusters. Bars with multiple colors denote admixed genomes.

Although six populations were initially sampled, the Bayesian analysis allowed for the identification of only two distinct genetic groups, because the highest Δ*K* value was achieved with *K *=* *2 (Fig.3). Thus, lowland populations L1, L2, and L3 were grouped in the first cluster (green, Fig.3) and upland populations U1, U2, and U3 in the second cluster (red, Fig.3), indicating a strong genetic structure that seems to be altitude related. We also noticed a lack of migrants between these two genetic groups, because no individual originally sampled at the lowland site was included in the upland cluster or vice versa. This structure was reinforced by the global *F*_ST_ = 0.21 and by the molecular analysis of variance, which showed greater variance between the upland and lowland sites (18.6%) than between populations (2.8%; Table2).

**Table 2:** Analysis of molecular variance (AMOVA; Excoffier et al. 1992) for *Bathysa australis* populations

Source of variation	Degrees of freedom	Sum of squares	Estimated variance	Percentage of variance (%)	*P*
Among sites (elevation band)	1	224.372	0.776	18.6	<0.001
Among populations (1-ha plots)	4	50.693	0.117	2.8	<0.001
Within populations (1-ha plots)	532	1746.016	3.282	78.6	<0.001
Total	537	2021.082	4.175		

Similarly, pairwise *F*_ST_ values were low between populations from the same altitude and high between populations from different altitudes, although all values were significant (Table3). The weak genetic structure observed between populations at the same altitude might result from the fine-scale spatial genetic structure. The gene flow among the upland populations was slightly higher than the gene flow among the lowland populations.

**Table 3:** Pairwise *F*_ST_ values between *Bathysa australis* populations in the upper triangle and geographic distance (km) in the lower triangle. All *F*_ST_ values are significant at *P *<* *0.01 (999 simulations)

	L1	L2	L3	U1	U2	U3
L1	–	0.048	0.049	0.242	0.206	0.209
L2	0.8	–	0.033	0.238	0.204	0.204
L3	0.9	0.1	–	0.240	0.203	0.204
U1	6.4	5.5	5.5	–	0.026	0.022
U2	6.3	5.5	5.5	0.2	–	0.014
U3	6.0	5.3	5.2	0.8	0.7	–

**Figure 4:**
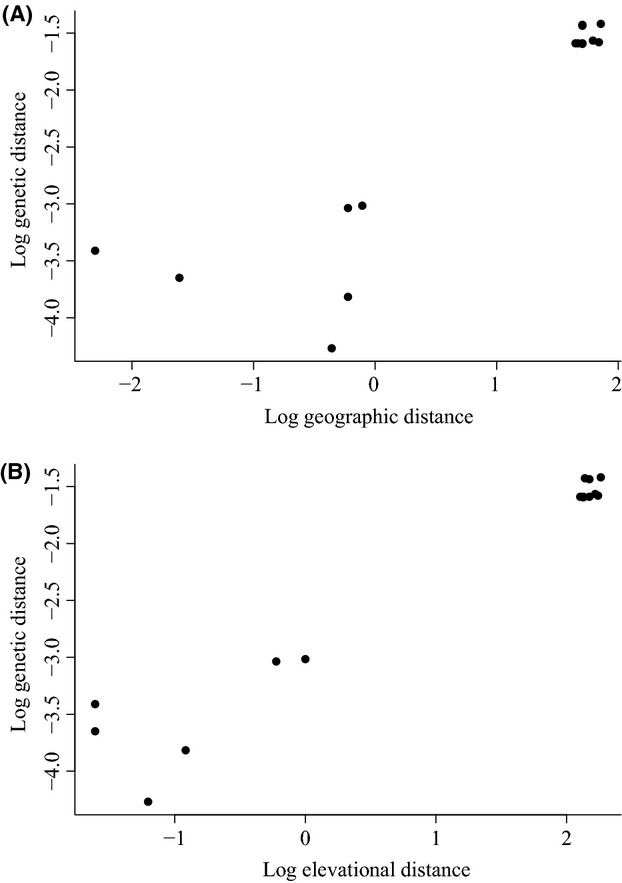
Isolation by distance and isolation by elevation patterns in *Bathysa australis*. Mantel correlations indicating population differentiation (pairwise *F*_ST_) as a function of A) geographic distance (*r* = 0.98, mantel*-P *=* *0.02) and (B) elevational distance (*r* = 0.99, mantel*-P *=* *0.02). However, when the partial Mantel test discounted the effect of altitude, the pattern of isolation by distance disappeared (*r* = −0.38, mantel-*P *=* *0.95). The pattern of isolation by elevation, on the other hand, proved consistent even after discounting the effect of geographic distance (*r* = 0.68, mantel-*P *=* *0.02).

We observed a significant positive correlation between genetic and geographic distance (*r* = 0.98, mantel*-P *=* *0.02; Fig.4A). However, when the effect of altitude was discounted by the partial Mantel test, this correlation disappeared (*r* = −0.38, mantel-*P *=* *0.95). The pattern of isolation by elevation, on the other hand, proved consistent even after discounting the effect of geographic distance (*r* = 0.68, mantel-*P *=* *0.02). This result suggests that geographic distance is not a factor influencing the genetic structure among sites. The elevation distance between sites seems to be a more relevant element. However, in order to confirm the correlation between elevation and genetic distance, we must increase our sampling effort to include *B. australis* populations from intermediate elevations (Fig.4B).

Within the altitudinal groupings, we found a correlation between genetic and geographical distance for the lowland site (*r* = 0.03, mantel-*P *=* *0.01), but not for the upland site (*r* = −0.027, mantel-*P *=* *0.78), suggesting that the strongest structure observed for the lowland site is a result of geographic distance. However, the partial Mantel test indicates that this correlation disappears again when the effect of altitude is discounted (*r* = 0.028, mantel-*P *=* *0.18). The same occurred with the correlation between genetic and elevational distance, so neither site showed an elevation effect (lowland: *r* = −0.025, mantel-*P *=* *0.79, upland: *r* = 0.016, mantel-*P *=* *0.3), which seems reasonable, because of the low altitudinal variation within sites.

### Between-altitude genetic diversity

Only the allelic richness differed between the lowland and upland sites (*P *=* *0.027). Contrary to our expectations, the lowland populations showed higher allelic richness (*A*_*R*_ = 7.169) than the upland ones (*A*_*R*_ = 6.591; Table1). In the same way, lowland populations had significantly higher rare allele counts than the upland ones (*P *=* *0.011; Table4). Exclusive allele counts, however, were not significantly different between sites (*P *=* *0.13), although the lowland populations had 20 more exclusive alleles than the upland populations (Table4).

**Table 4:** Exclusive and rare allele counts across lowland and upland *Bathysa australis* populations

Loci	Exclusive alleles		Rare alleles	
	Lowland (*N* = 140)	Upland (*N* = 129)	Lowland (*N* = 140)	Upland (*N* = 129)
BA02	3	0	4	3
BA14	8	3	7	1
BA15	9	9	10	9
BA22	2	4	3	3
BA24	4	2	5	4
BA25	3	4	14	13
BA26	3	5	9	8
BA28	5	0	5	3
BA30	20	10	18	7
Total	57	37	75^a^	51^b^

Different letters denote significant differences between lowland and upland populations (Wilcoxon paired test; *α *= 0.05).

### Bottleneck

None of the populations showed an excess of heterozygosity in relation to the expected at mutation–drift equilibrium. Therefore, there is no evidence for a historical bottleneck.

## Discussion

### Within-population genetic diversity

In natural populations of plants, a heterozygote deficiency at any locus can be driven by two mechanisms. The first is related to the mating system and possible deviations from panmixia. The second derives from the structuring of populations and the fixation of alleles. *B. australis* populations are prone to both mechanisms in the Serra do Mar mountain range. The facultative autogamy of this species, a result of its self-compatible mating system (Freitas and Andrich 2013), favors the occurrence of inbreeding. Although *B. australis* flowers are dichogamous, temporally separating the male and female functions in a flower, Freitas and Andrich (2013) suggested that geitonogamous crosses might predominate in *B. australis* populations in the Atlantic rain forest of Itatiaia, in the Serra da Mantiqueira mountain range. Besides, the spatially aggregated arrangement of *B. australis* individuals (*unpublished data*) may facilitate the occurrence of crossing events between relatives. The fine-scale spatial genetic structure has shown that nearby individuals are closely related.

Genetic structuring among populations is another way of increasing homozygote frequencies. *Bathysa australis* have shown a restricted gene flow between the lowland and upland populations, which, associated with random genetic drift, might promote the loss of some alleles and the fixation of others in different populations.

Although a heterozygote deficiency may have negative implications on population fitness, as a result of inbreeding depression, and may narrow a species’ capacity to cope with environmental changes by the loss of genetic variability (Keller and Waller 2002; Reed and Frankham 2003), it is a common feature in many plant populations (e.g., Hamrick et al. 1993; Hull-Sanders et al. 2005; Byars et al. 2009; Degen et al. 2013) and emerges naturally through the mechanisms cited above.

### Between-population genetic structure

The strong genetic structuring found between the lowland and the upland populations of *B. australis* in the Serra do Mar mountain range was expected, considering this species’ biological features. No dispersal vectors are known for the seeds of *B. australis*, which has been classified as an autochoric species. This means that its seeds travel just a few meters from the mother plant, producing a strong spatial genetic structure. As we have seen, with a distance of ∼10–20 m between them, individuals are more genetically similar than would be expected by chance. This feature generates the strong aggregate spatial distribution pattern that has been observed for this species in the Serra do Mar (*unpublished data*). It is thus possible that the restricted seed dispersal can lead to limited pollen dispersal by creating higher local tree densities, increasing the positive correlation between pollen and seed dispersal distances (Hardy et al. 2006). Moreover, fine-scale spatial genetic structure is mostly related to seed dispersal limitations, while the genetic structure on coarser scales is more related to pollen dispersal (Dick et al. 2008), as pollen flow is usually more extensive than seed flow (Petit et al. 2005).

*Bathysa australis* flowers are mostly pollinated by social bees and wasps (Freitas and Andrich 2013), small insects that usually fly over short distances (Dick et al. 2008). In closed canopy, small insects usually do not disperse beyond 300 m (Dick et al. 2008), although they can sometimes travel great distances (e.g., Janzen 1971; Dick et al. 2003). Despite that, it seems that these insects’ abundance is affected by altitudinal variation. Brito and Sazima (2012), working with the shrub species *Tibouchina pulchra* Cogn (Melastomataceae) at the same study site, observed that the availability of pollinators varied considerably with altitude and that the presence of effective pollinator bees was up to 200 times lower at the upland site in the Serra do Mar.

Animal pollination is strongly influenced by local density and flowering synchrony. High local densities, as we observed for *B. australis* populations at the study site, may restrict pollen dispersal by concentrating the pollinator’s resource on a single spot. Besides, synchrony leads to pollinator satiation, causing it not to visit other plants or to visit only very close ones (Dick et al. 2008). Furthermore, we observed a considerable asynchrony in the timing of flowering and fruiting phenophases between the upland and lowland *B. australis* populations (*personal observation*), which contributes to a limited pollen flow and, consequently, to a limited gene flow between these populations.

Finally, the mixed reproductive system of *B. australis* is another life-history feature that might strengthen the genetic structure of its populations. Self-compatibility may enhance inbreeding rates that, together with genetic drift, can lead to increased genetic divergence even between close populations (Young et al. 1996; Dick et al. 2008). Federov (1966) argued that this mechanism could promote speciation even in restricted spatial scales.

In the Serra do Mar mountain range, we found a marked genetic structure on a scale as small as 6 km. Even though *B. australis* is continuously distributed along the elevation gradient, we observed no gene flow between the lowland and upland populations, isolating these extreme groups. This creates a scenario for potential adaptation to the different conditions imposed by the elevation gradient. Behind such segregation, we have not found an isolation-by-distance pattern. Elevation difference was a relevant factor though, but we need further sampling effort to check its correlation with genetic distance.

### Between-altitude genetic diversity

There is a significant variation in the distribution patterns of genetic diversity within populations along altitudinal gradients (Ohsawa and Ide 2008). Other studies have shown peaks of diversity in higher (e.g., Gämperle and Schneller 2002), lower (e.g., Quiroga and Premoli 2007), and mid-elevations (e.g., Oyama et al. 1993; Byars et al. 2009). In addition, the lack of an altitudinal effect on genetic diversity is also frequently reported (e.g., Truong et al. 2007) and usually denotes an unrestricted gene flow among populations along the gradient. This does not seem to be the case for *B. australis*, which has a limited gene flow among the populations from the altitudinal extremes of the Serra do Mar. Still, we did not find higher genetic diversity in the upland populations, as would be expected, considering the optimum of environmental conditions for this species. On the contrary, the lowland populations exhibited greater allelic richness and higher exclusive and rare allele counts.

A possible explanation might be the existence of forces reducing upper mountain genetic diversity, as it would be the case for bottlenecks during forest movements (Ohsawa and Ide 2008). Quiroga and Premoli (2007) suggested that *Podocarpus parlatorei* genetic decline toward higher elevations reflected forest migration during glacial periods. In the same way, Ohsawa and Ide (2011) found that historical factors, rather than ecological ones, have primarily shaped intrapopulation genetic diversity distributions in montane species. The Serra do Mar mountain range formation began 65 million years ago (Hackspacher et al. 2004), and during this period forest ranges expanded and contracted several times following climatic oscillations. The Brazilian Atlantic montane forests that we know today emerged only 17,000 years ago (Behling 2008), and it is possible that since then some species originally from the lowland have started to climb the mountains. However, we did not find any evidence for a historical bottleneck in any population.

We could say that both populations might be already adapted to their local conditions, and each has its own set of optimum conditions, both experiencing high genetic diversity levels. However, the upland site might be more selective and the survival of individuals carrying new mutations would be compromised, keeping only alleles of high adaptive value. This may have led to the elimination of rare alleles in the upland populations, explaining the presence of more exclusive and rare alleles in the lowland, as well as its higher allelic richness. Further controlled transplant experiments might elucidate this question.

## Data Accessibility

Individual DNA sequences are available in GenBank (KF267877-KF267886). Microsatellite genotype data are available in DRYAD.
